# Commentary on: Clinical Use of Extracellular Vesicles in the Management of Male and Female Pattern Hair Loss: A Preliminary Retrospective Institutional Review Board Safety and Efficacy Study

**DOI:** 10.1093/asjof/ojac046

**Published:** 2022-07-08

**Authors:** Alfonso Barrera

**Affiliations:** Baylor College of Medicine, Houston, TX, USA

In recent years, the most common nonsurgical treatments to strengthen hair growth have included the use of minoxidil, finasteride, platelet-rich plasma (PRP), and adipose-derived stem cells, with modest but clear improvement in well over 50% of the patients (Video).

The extracellular vesicles (EVs) presented in Dr Sasaki's study are aseptic human bone marrow mesenchymal stem cell-derived EVs isolate from volunteered donors.^1^ It is an allogenic acellular treatment, so it has decreased immunogenicity.

The study presented included 22 female and 9 male patients who were treated with injections of EVs and followed for at least 6 months. Improvement of hair growth occurred in over 50% of the females and close to 80% of the males. I personally have not been involved with the use of EVs (exosomes); I have been using PRP and nano fat grafting and have found comparable results. It would be very interesting to see how long the improvement is maintained using EVs treatment when compared with using PRP or nano fat grafting.

## Supplementary Material

ojac046_suppl_Supplementary_Material
