# Supplementary material for: Commentary on: Clinical Use of Extracellular Vesicles in the Management of Male and Female Pattern Hair Loss: A Preliminary Retrospective Institutional Review Board Safety and Efficacy Study
Source: Aesthet Surg J Open Forum. 2022 Jul 8;4:ojac046. doi: 10.1093/asjof/ojac046 (PMC9336552; doi:10.1093/asjof/ojac046)
Supplement: ojac046_suppl_Supplementary_Material [file ojac046_suppl_Supplementary_Material.docx]

Hello there, my name is Alfonso Barrera. I’m a plastic surgeon in Houston Texas. Thank you for the kind invitation to participate in the video commentary on a scientific article titled “The Use of Extracellular Vesicles in the management of male and female hair loss” a preliminary IRB safety and efficacy study.

I have been doing hair transplantation for a long time, over 25 years, but as far as nonsurgical treatment for hair loss the most frequently treatments used to date include the use of minoxidil topical, finasteride orally, PRP platelet rich plasma injections, and nano fat grafting injections. In this study they are evaluating the use of extracellular vesicles.

What are extracellular vesicles? Extracellular vesicles are bioactive molecules released by all cells, and their main function is intercellular communication which may lead into cellular proliferation, cellular differentiation, angiogenesis, etc. In this study, they are using extracellular vesicles which are, which are obtained from aseptic human bone marrow, human bone marrow stem cells derived extracellular vesicles from volunteer donors.

So, it is allergenic, it is acellular, and it comes from packages of 1, 2, and 5 syringes mLs. Frozen packages. In this study they evaluate 22 females, and 9 males. Of the 22 females, 55% had an improvement in hair growth 36% had no improvement 9% got worse. In the males, there were 9 males evaluated, 78% had a significant improvement in hair growth and 22% of were stable, none of them got worse.

In my practice, I normally use PRP, platelet rich plasma; I draw in 50cc of, of peripheral blood and from there we come up with about 12cc of PRP and I recommend it every 12 every, every 6, every 6 months. And as far as the nano fat grafting, we extract about 12cc of fat, lipo aspirate and come up with about 5 cc of nano fat to inject and I recommend also every 6 months.

In this study they mention also that possibly the improvement that we get from PRP and nano fat grafting comes from the cells containing the injection and that might be true. What might be interesting to study is the length of the improvement that occurred with extracellular vesicles compared to the improvement that we get from PRP and nano fat grafting. I hope that helps and let me know if you have any questions.
